# Primary Congenital Glaucoma and the Involvement of *CYP1B1*

**DOI:** 10.4103/0974-9233.75878

**Published:** 2011

**Authors:** Kiranpreet Kaur, Anil K Mandal, Subhabrata Chakrabarti

**Affiliations:** Kallam Anji Reddy Molecular Genetics Laboratory, Prof. Brien Holden Eye Research Centre, L.V. Prasad Eye Institute, Hyderabad, India; 1 Jasti V Ramanamma Childrens Eye Care Center, Prof. Brien Holden Eye Research Centre, L.V. Prasad Eye Institute, Hyderabad, India

**Keywords:** *CYP1B1*, Gene, Glaucoma, Mutation

## Abstract

Primary congenital glaucoma (PCG) is an autosomal recessive disorder in children due to the abnormal development of the trabecular meshwork and the anterior chamber angle. With an onset at birth to early infancy, PCG is highly prevalent in inbred populations and consanguinity is strongly associated with the disease. Gene mapping of PCG-affected families has identified three chromosomal loci, *GLC3A*, *GLC3B* and *GLC3C*, of which, the *CYP1B1* gene on *GLC3A* harbors mutations in PCG. The mutation spectra of *CYP1B1* vary widely across different populations but are well structured based on geographic and haplotype backgrounds. Structural and functional studies on *CYP1B1* have suggested its potential role in the development and onset of glaucomatous symptoms. A new locus (*GLC3D*) harboring the *LTBP2* gene has been characterized in developmental glaucoma but its role in classical cases of PCG is yet to be understood. In this review, we provide insight into PCG pathogenesis and the potential role of *CYP1B1* in the disease phenotype.

## INTRODUCTION

Primary congenital glaucoma (PCG) is an ocular developmental anomaly that occurs due to the obstruction in the drainage of the aqueous humor outflow caused by the abnormal development of the trabecular meshwork (TM) and the anterior chamber angle.^1^ PCG manifests during neonatal or early infantile period and is characterized by elevated intraocular pressure (IOP), megalocornea, and Haab’s striae. Additional clinical features include corneal edema due to the elevated IOP that results in corneal haze, photophobia, ephiphora, and blepharospasm.^1^

## PREVALENCE OF PCG WORLDWIDE

The prevalence of PCG ranges from 1 in 1250 among the Gypsy population of Slovakia, 1 in 2,500 among the Saudi Arabians to 1 in 20,000 in the Western populations.^2^,^3^ The high rate of consanguinity among Slovakian Gypsies accounts for the increased prevalence of PCG. In Southern India, the prevalence of PCG is estimated at 1 in 3300 live births accounting for 4.2% of overall childhood blindness.^4^

### Genetics of PCG

The genetic basis of the disease was based on the segregation of PCG in families. For the first time, the endemic occurrence of PCG was observed in the Jewish population of Algiers. Later PCG was suggested to be an autosomal recessive disorder based on its inheritance pattern in a Swedish family.^5^ The high rate of concordance among monozygotic and disconcordance among the dizygotic twins also provided an additional genetic basis for PCG.^2^ The autosomal recessive mode of inheritance was also supported by the prevalence of consanguinity among the affected families and was the most common mode of inheritance reported in various studies.^5^ However, pseudominance has been observed in some pedigrees due to high rate of consanguinity and inbreeding in certain ethnic groups.^6^

### Candidate Loci in PCG

Currently, three chromosomal loci have been implicated in PCG on *GLC3A* at 2p21-22,^7^ *GLC3B* at 1p36.2-36.1,^8^ and *GLC3C* at 14q24.3.^9^

#### (a) GLC3A

The *GLC3A* locus was mapped based on a study on 17 Turkish families comprising 113 individuals.^7^ These families had a diagnosis of bilateral PCG within the first 6 months of birth without any other associated abnormality. Linkage analysis and further refinement suggested that 85% of the families showed homogeneity to the linked markers indicating that this region (2p21-22) was a major locus for PCG. This region was termed as *GLC3A*, where “GLC” was the designated symbol for glaucoma, “3” for congenital glaucoma and “A” as the first mapped locus in PCG.^7^

A similar study on seven PCG Slovakian Rom families based on homozygosity mapping and recombination events mapped the disease locus at an interval of 8 cM between D2S1788 and D2S1356. Genetic homogeneity in all these families was attributed to endogamy and high rate of inbreeding.^10^

#### (b) GLC3B

The second PCG locus was mapped to 1p36.2-36.1 through a study of eight PCG families that did not reveal linkage to *GLC3A*.^8^ A whole genome scan was conducted on these families with 126 short tandem repeat (STR) markers. The position of *GLC3B* was confirmed and the significant heterogeneity indicated the existence of another locus for PCG.^8^

#### (c) GLC3C

The third locus for PCG was mapped to 14q24.3 and was named as *GLC3C*.^9^ This study was carried out on a five-generation consanguineous family. On exclusion of *GLC3A* and *GLC3B* in this family, a whole genome scan was done with 235 STRs that led to the identification of homozygosity with only marker (D14S53) at 14q in the all the affected members. Further refinement revealed the homozygosity of the haplotype in all the affected members.^9^

## IDENTIFICATION OF CANDIDATE GENES

In 1995, Sarfarazi and co-workers established the critical region for the identification of the candidate gene on *GLC3A* using Yeast artificial chromosome (YAC) screening and radiation hybrid mapping.^7^ On the basis of sequence tagged sites (STS) and expressed sequence tag (EST) maps of the human genome, the position of various STR markers linked to *GLC3A* was refined.^11^ The critical region for *GLC3A* harbored two genes, human cytochrome P4501B1 (*CYP1B1*) and protein kinase interferon-inducible double stranded RNA-activated gene (*PRKR*), and a guanine nucleotide exchange factor for RAS (*hSOS1*). The splicing factor, arginine/serine-rich, 7 (*SFRS7*) gene was also mapped within the critical region by a BLAST search. As the pathophysiology of PCG was unknown and there was no prior evidence of an association of these genes to the disease phenotype, all of these were considered potential candidates. The coding regions of these genes were screened by direct sequencing that excluded *hSOS1* and *PRKR* as no variations were observed between them. However three different frameshift mutations were detected (two deletions and one insertion) in *CYP1B1* in three families that segregated in the affected individuals and were absent in 470 normal chromosomes.

The *GLC3B* (1p36.2-1p36.1) has been reported to be a GC-rich region and harbors a number of tumor suppressor genes.^12^ These genes are associated with various malignancies yet none of them have segregated with PCG. Similarly, seven genes are known to harbor the *GLC3C* region: Neurexin 3A, Nuclear Receptor ERRB2, KIAA0759, Glutathione Transferase Zeta 1, Maleylacetoacetate Isomerase, Serine Palmitoyl Transferase Subunit II and Alk B protein Homologue. However, none of these genes have been characterized so far in PCG.

### Cytochrome P4501B1 (*CYP1B1*; 2p21-2p22)

The *CYP1B1* gene harbors more than 70 mutations in PCG among various ethnic groups (http://www.hgmd.cf.ac.uk/ac/index.php). The cytochrome P450 (CYP450) is a superfamily of closely related heme proteins found throughout the phylogenetic spectrum of plants and animals. The term CYP450 arises from the presence of a heme group and their maximum absorption at 450 nm, which is unique for these proteins and serves as their signature.^13^ Cytochrome P450 proteins have an average mass of approximately 50 kDa. These are mostly membrane bound, anchored to the endoplasmic reticular membrane or the inner mitochondrial membrane with few soluble forms found in bacteria.^14^ Structurally they consist of a hydrophobic amino terminal region, a proline-rich region, a carboxyl terminal portion that consists of a set of conserved core structures and signature sequences and a substrate binding region, which is a less conserved region between the hinge region and the conserved core structure.^14^

The cytochrome P450 1B1 (*CYP1B1*) is the only member in the CYP1B subfamily and shares 40% homology with two members in the CYP1A subfamily (CYP1A1 and CYP1A2).^15^ *CYP1B1* has the highest catalytic activities toward several polycyclic aromatic hydrocarbons that are the most potent inducers of mammary tumors and lung cancers.^16^ Apart from these exogenous compounds *CYP1B1* is also involved in the metabolism of endogeneous steroids such as 17β estradiol. A significantly higher expression with elevated 4-hydroxy estradiol production has been found in various tumor tissues including breast, uterus, prostate, lungs, than in the normal tissues.

It has been demonstrated that single nucleotide polymorphisms (SNPs) in *CYP1B1* (R48G, A119S, and V432L) exhibit altered kinetics for hydroxylation of estradiol.^17^,^18^ These SNPs show significant associations with various cancers with respect to the genotypic and allelic frequencies in patients than controls.^19^–^21^ Thus alterations in these residues are likely to affect the activity of the enzyme and may influence the susceptibility of individuals toward endogenous and exogenous carcinogens.^22^

### *CYP1B1* expression in Ocular Tissues

*CYP1B1* protein expresses in various human ocular tissues including cornea, ciliary body, iris, and retina. However, no expression has been identified in TM. Interestingly, an increased *CYP1B1* expression has been identified in fetal eyes compared to the adult eyes. This observation makes it tempting to speculate that *CYP1B1* in the eye metabolizes an important substrate that plays a key role in the development and maturation of ocular tissues.^23^ However, an entirely opposite scenario has been observed in the mouse ocular tissues. For example, a study that evaluated the spatio-temporal expression of *CYP1B1* in mouse eye ontogeny revealed that the expression increases with age.^24^ Based on the observations of these two studies, it can be suggested that *CYP1B1* expression pattern is different in these two species.

### Use of *CYP1B1* Knockout Mice to Determine Its Role in PCG Pathogenesis

To investigate the role of *CYP1B1* in PCG, *CYP1B1* knockout (*CYP1B1^-/-^*) mice were generated by targeted gene disruption in embryonic stem cells. However, gross examination of the eyes of these *CYP1B1^-/-^*mice neither showed any evidence of glaucoma nor any systemic abnormalities suggesting that *CYP1B1* is not required for mammalian development.^25^

Similarly, the *CYP1B1^-/-^*mice created by Libby and co-workers (2003) showed no gross abnormalities with their IOP being indistinguishable from the wild mice.^26^ However, histological and electron microscopic analysis revealed abnormalities in the eyes of *CYP1B1^-/-^*mice such as small or no Schlemm’s canal, basal lamina extending from the cornea over the TM and attachments of iris to TM and peripheral cornea. These observations indicate that even though the *CYP1B1^-/-^*mice did not exhibit features of classical PCG, the anterior chamber angle abnormalities indicated the potential role of *CYP1B1* in the development of these ocular structures.^26^

### Potential Role of *CYP1B1* in development through retinoic acid-mediated signaling

*CYP1B1* has been shown to metabolize the two step oxidative synthesis of retinoic acid (RA) from retinol. RA is a ligand for various nuclear receptor proteins and is known to regulate morphogenesis. The two step oxidative synthesis of RA can be metabolized by various retinaldehyde dehydrogenases (RALDH) such as RALDH-1, RALDH-2, and RALDH-3. In order to correlate the RA synthesis by *CYP1B1*, Chambers and co-workers (2007) studied regions in chicks, which are rich in RA production and *CYP1B1* synthesis but are deficient in RALDH expression.^27^ These authors studied the ectopic expression of *CYP1B1* and in turn its effect on the transcription of developmental genes, which are known to be regulated by RA. Furthermore, they found that ectopic expression of *CYP1B1* resulted in the downregulation of two such developmental genes, i.e. Shh (sonic hedgehog homolog of drosophila) that regulates the vertebrate organogenesis and *Nkx6.1* (NK homeobox family 6), involved in the development of beta cells in endocrine pancreas.^27^ Based on these findings, it was suggested that *CYP1B1* might play an important role in the development via RA-mediated signaling pathways.

### Mutation Spectrum of *CYP1B1* Worldwide

More than 70 distinct mutations (http://www.hgmd.cf.ac.uk/ac/index.php) in *CYP1B1* have been described in PCG indicating the genetic heterogeneity of the condition [Table 1]. The prevalence of *CYP1B1* mutations ranges from 20% in Japanese,^28^ 33.3% in Indonesians,^29^ 44% among Indians,^30^ 50% among the Brazilians^6^ to almost 100% among the Saudi Arabians^31^ and Slovakian Gypsies.^32^ The relatively higher prevalence of these mutations in the latter two populations could be attributed to consanguinity and inbreeding.

**Table 1:** Worldwide distribution of *CYP1B1* mutations

Nucleotide position	Amino acid change	Type of mutation	Exon	Population	Reference
g.3775insA	-	Frameshift	II	India	33
g.3860C>T	Q19X	Nonsense	II	Brazil	6
g.3928delG	-	Frameshift	II	India	Our study
g.3905-3927del23	-	Frameshift	II	India	34
g.3964delC	-	Frame shift	II	Japan	28
g. 3972 C>T	A56V	Missense	II	India	Our study
g.3976G>C	W57C	Missense	II	Hispanic	35
g.3896G>A	W57X	Nonsense	II	Brazil	6
g.3988delA	-	Frameshift	II	Iran	36
g.4035 T>C	L77P	Missense	II	Saudi Arabia	31
g.4046 T>A	Y81N	Missense	II	India	Our study
g.4048C>A	Y81X	Nonsense		Iran	36
g.4055 G>T	V84F	Missense	II	India	Our study
g.4148 G>C	A115F	Missense	II	India	37
g.4154C>T	R117W	Missense	II	Turkey	38
g.4200 T>G	M132R	Missense	II	India	37
g.4236 A>C	Q144P	Missense	II	India	37
g.4236A>G	Q144R	Missense	II	India, Spain	39, 40
g.4322G>A	E173R	Missense	II	Iran	36
g.4339delG	-	Frameshift	III	Morocco	41
g.4380A>T	D192V	Missense	II	Japan	28
g.4383C>T	P193L	Missense	II	India	37
g.4397G>A	V198I	Missense	II	Japan	28
g.4410C>A	A202D	Missense	II	Iran	36
g.4449G>T	S215I	Missense	II	Indonesia	29
g.4490G>A	E229K	Missense	II	Iran, India, France, Turkey	34,36,38,42-46
g.4499G>C	G232R	Missense	II	France	45
g.4520 A>C	S239R	Missense	II	India	34
g.4547C>T	Q248X	Missense	II	France	45
g.4611-4619dup9	-	Frameshift	II	Iran	36
g. 4645 C>A	C280X	Nonsense	II	India, Japan	28, Our study
g.4646G>T	E281X	Frameshift	II	Turkey	35
g.4673-4674insC		Frameshift	II	Iran	36
g.4677A>G	D291G	Missense	II	Iran	36
g.4763G>T	V320L	Missense	II	Japan	28
g. 4776insAT		Frameshift	II	Japan	28
g.4791G>T	G329V	Missense	II	Japan, Iran, Turkey	28,36,38
g.4791G>T	G329D	Missense	II	Australia	47
g.4793G>T	A330F	Missense	II	Japan	48
g.4793 G>A	A330T	Missense	II	India	Our study
g.7900 C>T	Arg355Stop	Nonsense	III	Turkey, India	49, Our study
g. 7900-7901 del2	-	Frameshift -	III	Indian	37
g.7927G>A	V364M	Missense	III	Japan, Indonesia	28,29
g.7934G>T	G365W	Missense	III	USA	35
g.7934delG	-	Frameshift	III	Iran	36
g.7939C>T	R368C	Missense	III	Iran	36
g.7940G>A	R368H	Missense	III	India, SaudiArabia, Brazil, Turkey, Kuwait, Iran	6,31,33,34,36,38,39,42,50
g.7957G>A	D374N	Missense	III	Saudi Arabia	31
g.7971C>T	P379L	Missense	III	Turkey	35
g.7996G>A	E387K	Missense	III	Brazil, France	6,45
g.8006G>A	R390H	Missense	III	India, Iran, France,	36,37,46
g.8005 C>A	R390S	Missense	III	Saudi Arabia	31
g.8005 C>T	R390C	Missense	III	India	34
g.8033T>G	I399S	Missense	III	France	45
g.8035C>T	P400S	Missense	II	Australia	47
g.8037-8046 dup10	-	Frameshift	III	Brazil, USA, British, Turkey, France, Germany, UK, Costa Rica, Iran, Australia	6,35,36,45,47,49,52,53
g.8147 C>T	P437L	Missense	III	Turkey, Brazil, India	6,34,35
g.8148-8152 del5	-	Frameshift	III	India	Present study
g.8162C>G	P442R	Missense	III	Iran	36
g.8165C>G	A443G	Missense	III	Brazil, France	6,49
g.8168G>A	R444Q	Missense	III	Japan	28
g.8182delG	-	Frameshift	III	Brazil	6
g.8214-8215del2	-	Frameshift	-III	Brazil	6
g.8227T>C	S464P	Missense	III	India	Present study
g.8234 G>A	G466D	Missense	III	India	34
g.8242C>T	R469W	Missense	III	Saudi Arabia, Turkey, Iran	31,35,36
g.8333A>G	E499G	Missense	III	Japan	28
g.8341delA	-	Frameshift	III	Iran	36
g.8354-8373delA	-	Frameshift	III	Iran	36
g.8393 A>G	Asn519Ser	Missense	III	India	Present study

The majority of these mutations are missense mutations affecting the amino acid residues located either in the hinge region or the conserved core structures in the cytosolic region [Table 2]. These mutations, therefore, are expected to interfere with fundamental properties of the protein such as folding, heme binding, substrate accommodation, and interaction with the redox partner.^14^ As these mutations have been identified throughout the gene, there are no major hot spot regions in the *CYP1B1*.

**Table 2:** Distribution of different types of *CYP1B1* mutations worldwide

Population (n)	Type of mutation					Cases without *CYP1B1* mutation
	Missense	Nonsense	Deletion	Duplication	Insertion	
Saudi Arabia (n=62)^31^	55 (88.7)	-	2 (3.2)	-	-	5 (8.1)
Brazil (n=52)^6^	5 (9.6)	3 (5.8)	16 (30.8)	4 (7.8)	-	24 (46.1)
Iran (n=104)^36^	81 (77.9)	1 (0.96)	4 (3.84)	3 (2.9)	1 (0.96)	14 (13.5)
Australia (n=37)^47^	8 (21.6)	1 (2.7)	1 (2.7)	1 (2.7)	-	26 (70)
Japan (n=65)^28^	10 (15.4)	1 (1.5)	1 (1.5)	-	3 (4.6)	50 (76.9)
Turkey (n= 35)^38^	13 (37.1)			1 (2.9)	1 (2.9)	20 (57.1)
Ecuador (n=15)^54^	2 (13.3)	-	1 (6.7)	-	-	12 (80)
Kuwait (n=17)^50^	11 (64.7)	1 (5.9)	-	-	-	5 (29.4)
Indonesia and Europe (n=21)^29^	5 (23.8)	1 (4.8)	2 (9.5)	-	-	13 (61.9)
Morocco (n=32)^41^	9 (28.1)	-	4 (12.5)	-	-	19 (59.4)
France (n=31)^45^	6 (19.4)	9 (29.0)	2 (6.5)	-	-	14 (45.2)
Mexico (n=12)^55^	4 (33.3)	-	-	1 (8.3)	-	7 (58.3)
US and Brazil (n=21)^52^	2 (9.5)	-	2 (9.5)	2 (9.5)	-	15 (71.4)
Egypt (n=10)^56^	3 (30)	-	-	-	-	7 (70)

Figures in parentheses are in percentage

### Prevalent Mutations in *CYP1B1*

Among the various *CYP1B1* mutations, the G61E was the most prevalent mutation in 69.3% (43/62) of Saudi Arabians and 29% (30/104) of Iranian patients [Table 3]. Apart from G61E, the R390H and R368H mutations were also identified with a higher frequency (20.1% and 10.6%, respectively) in Iranian patients. In the Saudi Arabian population, 44.2% (19/43) of the patients with G61E did not manifest the disease at presentation, indicating the incomplete penetrance of this mutation.^31^ In the Iranian population, 26/29 cases with G61E were bilateral with an age of onset within 3 months of life and raised IOP.^36^

**Table 3:** Prevalent *CYP1B1* mutations in different PCG populations

Prevalent mutation	Population	Number of patients screened	Cases with prevalent mutation	References
G61E	Saudi Arabia	62	43 (69.3)	31
	Iran	104	30 (29)	36
	Turkey	35	5(14.3)	38
	Kuwait	17	9 (52.9)	50
4339delG	Morocco	32	9 (28.1)	41
4340delG	Brazil	52	12 (23)	6
R444Q	Australia	37	2 (5.4)	47
	Japan	65	3 (4.6)	28
V364M	Japan	65	4 (6.2)	28
	Indonesia and Europe	21	4 (19)	29
8037-	USA + Brazil	21	2 (9.5)	52
8046dup10				
R368H	India	138	25 (18)	34
E387K	Slovakia	20	20 (100)	32

Figures in parentheses are in percentage

E387K was the only mutation identified in all the 20 PCG cases among the Slovakian gypsies suggesting a single ancestral mutational event [Table 3]. The common haplotype background of the patients harboring this mutation further confirmed the founder effect of E387K mutation in this population.^32^

In the Brazilian population, 4340delG was the most predominant mutation identified in 23% (12/52) of cases [Table 3]. All patients with 4340delG had bilateral disease with an age at onset within the first month of life and IOP ranging from 25 to 55 mmHg.^6^

The R368H mutation was the most predominant *CYP1B1* mutant allele identified in PCG among Indian cases [Table 3]. However, clinically there was no association of severity of the disease phenotype with this mutation.^37^,^42^

### Clustering of Prevalent *CYP1B1* Mutations on Common Haplotype Backgrounds

Haplotypes generated with five intagenic SNPs (R48G, A119S, V432L, D449D and N453S) in *CYP1B1* in different populations indicated that the “C-G-G-T-A” was the most prevalent haplotype amongst PCG cases harboring *CYP1B1* mutations, while the “G-T-C-C-A” was the most common haplotype among the controls.^39^ Most of the common mutations in different populations were found to cluster on the “C-G-G-T-A” haplotype and thus this must have been an ancient haplotype. An extensive haplotype analysis supplemented by evolutionary insights suggested that the clustering of mutations from different geographical regions on the same haplotype background is suggestive of their common founder effects. Also, the occurrence of the same mutation on similar haplotype backgrounds in different geographical regions is probably due to human migration.^39^ This phenomenon has also been observed in cases with *CYP1B1* mutations in primary open angle and primary angle closure glaucomas.^57^

### Interaction of *CYP1B1* with Other Genes

Myocilin (*MYOC*), a candidate gene for juvenile and adult onset forms of primary open angle glaucoma, may have been involved with *CYP1B1* through a digenic mechanism in a glaucoma family of East Indian (Guyanese) origin. It has been suggested that congenital glaucoma and juvenile glaucoma are allelic variants of *CYP1B1* and that *CYP1B1* and *MYOC* might act through common biochemical pathways with *CYP1B1* acting as a modifier for *MYOC*.^58^ Digenic interactions of *MYOC* and *CYP1B1* alleles have also been reported in cases of PCG.^59^

*CYP1B1* has also been reported to interact with tyrosinase (*Tyr*), a candidate gene for ocular albinism that codes for a protein which converts tyrosine to L-dopa, a precursor of catecholamines and an important regulator in development. *Tyr* has been identified as a modifier in iridocorneal angle defects present in *CYP1B1* knockout mice. It has been observed that mice lacking both *CYP1B1* and *Tyr* had severe iridocorneal angle malformations than those lacking only *CYP1B1*.^26^ Also some albinos are reported to have anterior segment dysgenesis and congenital glaucoma suggesting the possible role of *Tyr* in congenital glaucoma. It has been hypothesized that *Tyr* may affect the angle development through modulation of L-dopa as treatment of L-dopa was found to prevent the severe angle dysgenesis present in mice lacking both *CYP1B1* and *Tyr*.

However, *TYR* did not show any association with human PCG patients. Bidinost and co-workers (2006) conducted a genome wide scan on 97 individuals from 17 Saudi Arabian families of whom, 58 had homozygous or compound heterozygous mutations in *CYP1B1*.^60^ The genome wide scan did not show any significant evidence for a co-segregating locus and resequencing of *TYR* did not reveal any mutations in PCG. Thus, it was suggested that *TYR* was not a modifier for *CYP1B1* mutations in humans and the possible role of *TYR* in normal human eye development may be different from that in the mouse.^60^

### *CYP1B1* Mutations with PCG pathogenesis

The exact role of *CYP1B1* in the development of eye and its association with PCG is not known. Various in silico and *In vitro* studies have been carried out to determine the impact of the mutations in *CYP1B1* on the structure and function of the protein. The findings of these studies can help in a better understanding of the association of *CYP1B1* with the disease pathogenesis.

Recently, Hollander and co-workers tried to correlate *CYP1B1* mutations with the degree of angle dysgenesis observed histologically and disease severity in terms of age at diagnosis and difficulty in controlling IOP in six congenital glaucoma patients.^61^ Their findings suggested that *CYP1B1* mutations could be classified based on histological findings, which may be used to correlate these mutations with disease severity.

Certain *CYP1B1* mutations have been analyzed *in-silico* for their possible impact on the protein structure and function. Comparative modeling of the human *CYP1B1* using the X-ray structure of CYP2c9 as a template along with molecular dynamics simulations revealed several structural differences that would potentially impact the functional domains.^62^

*In vitro* studiesto determine the effect of *CYP1B1* mutations on the stability and function of the protein was carried out by Jansson and co-workers.^63^ These authors studied the effect of two missense mutations (G61E and R469W) on the stability and enzymatic activity of *CYP1B1*. It was observed that G61E mutant had lost 60% of its stability, while R469W retained about 80% of the stability compared to the wild type. The effects of the mutants on the function of protein were further determined by an enzymatic assay that further confirmed their decreased metabolic activity (50-70%) for all the substrates when compared to the wild protein.^63^

Similarly, Bagiyeva and co-workers compared the enzymatic activity of the two other mutant (R117W and G329V) proteins with the wild type. While there was no apparent difference in the expression levels of wild type and mutant proteins, there was a decreased enzymatic activity of mutant proteins compared to the wild type. This was attributed to the slow *CYP1B1* traffic through ER in cases of mutant *CYP1B1* that further contributed to the lower enzyme activity and that could lead to PCG pathogenesis.^38^

### Hypothesis for the Possible Role of *CYP1B1* in PCG Pathogenesis

Although the exact function of *CYP1B1* protein in the eye is still unclear but as it is a mono-oxygenase, the following scenarios may be expected for its role in the development of the eye.

*CYP1B1* is involved in the generation of some morphogen that plays an important role in the development of the TM and other components in the outflow system by regulating the spatial and temporal expression of genes controlling anterior chamber angle development. Hence mutations in *CYP1B1* might result in the absence of the morphogen, which in turn alters the expression of genes.^14^

Alternatively, *CYP1B1* eliminates some active morphogen and prevents its signal capacity from dispersing beyond the specific cells upon which it must act. Hence the mutations in *CYP1B1* might result in the accumulation of this metabolite producing toxic effects, which in turn may lead to developmental arrest.^14^

Although the absence of the orthologous enzyme in mouse (knockout mouse) did not show any evidence of glaucoma, this might be due to the less sensitive methods having been used to evaluate glaucomatous changes in the mouse. In addition, the mouse phenotype may differ from humans, since the anterior chamber angle has undergone evolutionary changes as evident from the differences in the TM in humans and higher apes as opposed to a reticular type meshwork in lower organisms.^14^,^64^

A recent study carried by Rojas and coworkers^65^ attempted to determine the pathogenic mechanisms of the disease by comparing trabeculectomy specimens of congenital glaucoma patients with normal human eyes, both at the histological and ultrastructural levels. They found accumulation of amorphous material in the TM, insertion of iris in TM, bulky endothelial cells in Schlemms canal, and increase in the size of trabeculae. Based on these findings, it was concluded that abnormalities in the TM structure could result in the disease phenotype.^65^

### Genotype Phenotype Correlation with Respect to *CYP1B1* Mutations

Despite a number of PCG-associated mutations in *CYP1B1*, little evidence exists of direct correlation of the mutant genotype with the disease phenotype. Such an association has been demonstrated in a Brazilian population where the most prevalent mutation (4340delG), identified in 21 out of 52 (20.2%) of the PCG cases, exhibited a very severe phenotype.^6^ The clinical evaluation of 12 individuals with this mutation revealed that all were bilateral cases, with an early onset of the disease (≤1 month of life) and with a maximum IOP ranging between 25 and 55 mmHg. These patients had a poor response to surgery when compared to those who did not have this mutation.^6^

On the other hand there are some reports about the variable expression of the disease phenotype. Four affected individuals in an American PCG family were found to be compound heterozygotes for the E387K and 268del SNF. But only two of them manifested a severe form of the disease with IOP of 25 and 28 mmHg in the right and left eyes, respectively, and corneal edema, whereas, the other two did not show any symptoms until their mid-teens.^52^

Two PCG patients along with a JOAG case from Costa Rica were reported to harbor the same homozygous (g.8037-8046dup) mutation.^53^ These patients had a maximum IOP of 22 and 24 mmHg, respectively. The former patient underwent two surgeries in the right eye and six surgeries in the left eye, whereas the latter patient underwent two surgeries in both eyes. All the three cases did not harbor any *MYOC* mutation. The presence of the same mutated allele resulting in manifestation of the disease symptoms at different age indicates the possible relationship between the genotype and the environmental factors.

A study from India on 146 Indian PCG patients tried to correlate the genotype of patients screened for six different mutations (Ins376 A, P193L, E229K, R390C, G61E and R368H) to disease severity. Accordingly, a severity index was prepared that included corneal diameter, IOP, cup to disk ratio, corneal clarity, and last recorded visual acuity.^42^ Cases with frameshift mutation (ins376A) had the worst phenotype followed by those with homozygous R390C mutation. In addition 80% of the cases with E229K, 72% with R368H, 66.7% with G61E and 62.5% with P193L exhibited a severe phenotype in at least one eye.^42^

A study on the Arab-Bedouin PCG cases determined the prognostic factors for surgical outcomes in cases that underwent intervention within the first 3 months of birth.^66^ The initial measure of outcome was based on surgical intervention, which was considered successful when normal IOP (<21 mmHg) was attained without antiglaucoma medication. The final outcome was considered a success when IOP was between 5 and 21 mmHg at the end of 2 year follow-up without anti-glaucoma medication irrespective of the number of surgical interventions. It was observed that among the patients with a failure in the first outcome, the initial IOP was higher (40.47 ± 11.06) compared to the group with lower IOP (34.14 ± 4.95) where it was a success.^68^ However, there was no association with the mean age at intervention, corneal diameter, and cup to disk ratio with the final outcome.^66^

## CONCLUSIONS

PCG is a clinical and genetically heterogeneous condition with *CYP1B1* as the major candidate gene. PCG-associated *CYP1B1* mutations have been reported worldwide in different populations with varying prevalence. However, *CYP1B1* alone cannot explain the overall genetic contributions in PCG. The exact role of this gene in the pathophysiology of the disease remains unknown. However, various *In vitro* and *in-silico* studies have demonstrated the pathogenic nature of the identified mutations. While several studies have been undertaken on PCG and *CYP1B1* but there is a dearth of studies in understanding the genetic basis of cases without *CYP1B1* mutations. Some attempts in understanding the molecular involvement of candidate genes such as *MYOC* and *FOXC1* have not indicated any major involvements.^59^,^67^ The mapping of a new locus (*GLC3D*) on 14q24, which is distal to *GLC3C* has been characterized to harbor mutations in the *LTBP2* gene in developmental glaucomas.^68^ While this is a promising development but its role in classic cases of PCG and those devoid of mutations in *CYP1B1* would be interesting.

Since PCG is a congenital disorder, an early and reliable diagnosis is vital, so that appropriate and prompt medical and/or surgical intervention can be initiated. This could prevent unwanted vision loss and also reduce the burden of childhood blindness. Some attempts in understanding the molecular basis through genome wide association studies and whole genome resequencing approaches are underway and may provide valuable insight regarding the underlying mechanisms. A comprehensive understanding of PCG-associated mutations in candidate genes would be helpful in developing a reliable diagnostic method for screening in the predisposed families that would eventually aid in predictive testing and better prognosis.
